# Autoimmune/Inflammatory Arthritis Associated Lymphomas: Who Is at Risk?

**DOI:** 10.1155/2016/8631061

**Published:** 2016-06-27

**Authors:** Sujani Yadlapati, Petros Efthimiou

**Affiliations:** ^1^Rheumatology Division, New York Methodist Hospital, Brooklyn, NY 11215, USA; ^2^Weill Cornell Medical College, New York, NY 10065, USA

## Abstract

Specific autoimmune and inflammatory rheumatic diseases have been associated with an increased risk of malignant lymphomas. Conditions such as rheumatoid arthritis (RA), primary Sjögren's syndrome (pSS), systemic lupus erythematosus (SLE), dermatomyositis, and celiac disease have been consistently linked to malignant lymphomas. Isolated cases of lymphomas associated with spondyloarthropathies and autoinflammatory diseases have also been reported. Direct association between autoimmunity and lymphomagenesis has been reinforced by large epidemiological studies. It is still uncertain whether disease specific determinants or phenotypic or treatment related characteristics increase likelihood of lymphomagenesis in these patients. For example, recent literature has indicated a positive correlation between severity of inflammation and risk of lymphomas among RA and Sjögren's syndrome patients. It is also debated whether specific lymphoma variants are more commonly seen in accordance with certain chronic autoimmune arthritis. Previous studies have revealed a higher incidence of diffuse large B-cell lymphomas in RA and SLE patients, whereas pSS has been linked with increased risk of mucosa-associated lymphoid tissue lymphoma. This review summarizes recent literature evaluating risk of lymphomas in arthritis patients and disease specific risk determinants. We also elaborate on the association of autoimmune arthritis with specific lymphoma variants along with genetic, environmental, and therapeutic risk factors.

## 1. Introduction 

Malignant lymphomas, particularly non-Hodgkin's lymphomas (NHL), are among the most commonly diagnosed malignancies in the United States [1]. One of the established associations is the occurrence of lymphomas in a setting of chronic inflammation. In the past decades, a higher incidence of lymphomas has been reported in patients with a range of chronic autoimmune and inflammatory rheumatic diseases. These ailments include rheumatoid arthritis (RA), systemic lupus erythematosus (SLE), primary Sjögren's syndrome (pSS), dermatomyositis, and celiac diseases. Isolated cases of malignant lymphomas have also been reported in patients with spondyloarthropathies such as psoriatic arthritis and ankylosing spondylitis, as well as chronic autoinflammatory arthritis such as Still's disease and systemic juvenile idiopathic arthritis. A higher predilection for malignant lymphomas has been seen among these patients when compared to the general population. This association has been widely established by a multitude of population based epidemiological studies across the world. However, the level of risk reported across the globe in autoimmune arthritis patients is not universal. This in turn raises the issue of whether the risk of lymphoma applies equally to all the patients diagnosed with the abovementioned rheumatic conditions.

Over the past decade, the pathogenesis of lymphoma in autoimmune diseases has been vastly explored; however, the exact biology behind the process is yet to be completely understood. Several plausible hypotheses have been suggested based on observational studies. These studies have proposed an increased risk of lymphomas in patients with immune dysregulation, patients receiving immunosuppressive drugs, or those who have been exposed to risk factors such as smoking or unknown environmental factors. It is also imperative to understand the variability in lymphomagenesis in rheumatic patients with different racial and genetic profiles. Another reason behind the inconsistency in risk estimates is that earlier and smaller studies reported higher risk levels compared to the more recent population based studies which have been larger [2, 3]. Data from the recent studies also supports the notion that increased lymphoma risk may be restricted to one or two specific lymphoma subtypes and to patients with particular determinants of immune mediated disease. One such established association is the relationship between disease intensity in RA and development of lymphoma, diffuse large B-cell lymphoma (DLBCL) in particular [4].

In this review, we aim to summarize the risk of lymphomas in specific autoimmune/inflammatory arthritis. Alternatively, we elaborate on the association of autoimmune arthritis with specific lymphoma variants along with disease specific, genetic, environmental, and therapeutic risk factors that can potentially be implicated in the pathogenesis of lymphoma.

## 2. Methodology

Online search was performed using the keywords such as “autoimmune arthritis” “inflammation”, “lymphomagenesis”, and “lymphoma”. This paper was written after reviewing full-text articles written in English found on PubMed. Inclusion criteria for studies included in this review are those that have been published in the past 25 years. Studies included to show an association between autoimmune arthritis and lymphomagenesis met the following criteria: (1) case control/cohort study; (2) autoimmune arthritis, specifically rheumatoid arthritis (RA), systemic lupus erythematosus (SLE), Sjögren's syndrome, dermatomyositis, polymyositis, ankylosing spondylitis, and psoriatic arthritis; (3) lymphoma (NHL or HL) as an outcome of interest; (4) relative risk (RR), standardized incidence ratio (SIR), odds ratio (OR), or hazard ratio (HR) with 95% confidence intervals (CIs). Articles have been included in this review on the basis of their relevance.

## 3. Risk of Autoimmune Arthritis Associated Lymphoma 

Traditionally, lymphomas are classified as non-Hodgkin's lymphoma (NHL) and Hodgkin's lymphoma. NHL accounts for almost 90% of all cases whereas Hodgkin's lymphoma comprises the remaining 10% [5]. NHL is further classified into T-cell and B-cell neoplasm based on the cell of origin. Here, we bring forth several population based studies evaluating increased risk of both NHL and HL in patients with a range of rheumatologic conditions (Table 1). It is evident that the magnitude of risk varies considerably among the studies. It is important to note that risk of lymphoma reported in each study is subject to variation depending on the population size and the population being studied. Earlier studies with smaller study population have reported higher risk estimates with a wide confidence interval when compared to the larger population based studies that have been published in the recent years. As per the published literature, the highest relative risk of lymphoma is associated with Sjögren's syndrome followed by RA and SLE. Relatively lower level of risk has been seen in patients with inflammatory myositis. Minimal risk if at all has been seen in patients with ankylosing spondylitis and psoriasis. Once again, this emphasizes the role of disease specific risk. A variation in the level of risk has been seen among patients in USA and countries such as Denmark or Sweden. This in turn raises the issue of population specific risk of lymphoma in these rheumatic conditions and implications of genetic and environmental influences on lymphomagenesis.

## 4. Arthritis Specific Lymphoma Variants

With newly emerging data from large pooled studies, it has become exceedingly evident that specific lymphoma subtypes are more commonly associated with particular types of autoimmune or inflammatory arthritis (Table 2). These NHL subtypes arise from singular stages of lymphocyte maturation. Therefore, association between specific autoimmune arthritis and corresponding NHL subtypes could lead to a superior understanding of mechanisms involved in lymphomagenesis. And, among these subclasses of arthritis patients, individual risk factors predispose the patient to a higher likelihood of developing lymphoma. This area has been further elaborated in the later sections of this paper.

A well known example of this is the strong association between patients with rheumatoid arthritis and diffuse large B-cell lymphoma (DLBCL). In a study conducted by Baecklund and associates, 67% of all RA/NHL cases (22 patients among 33 RA/NHL patients) were DLBCLs, compared to 30–40% of NHLs in the general population [6]. Similarly, in a case controlled study of 378 RA associated lymphomas, a high proportion of DLBCL was noted more than any other lymphoma subtype [4]. Similar findings were also described in a population based case control study in Denmark and Sweden enrolling 3055 NHL patients and 3187 matched control subjects [7]. Additionally, a stronger association of DLBCL with high disease activity in RA patients was also reported [7]. More comprehensive description of these RA associated DLBCLs has revealed that vast majority are of the nongerminal center (GC)/activated B-cell (ABC) subtype [8, 9]. Further evidence for the role of B cells in the development of RA-DLBCL came from a study investigating the expression of APRIL, a cytokine essential for B-cell proliferation and development [10]. In this study, expression of APRIL was investigated in DLBCL tissue obtained from RA and SLE patients and compared to tissue obtained from DLBCL patients with no obvious inflammatory disease [10]. Higher expression of APRIL was detected in a subset of RA patients with high disease activity compared to RA patients with low disease activity. Similarly, higher expression of this cytokine was seen in SLE and RA patients when compared to those without an inflammatory disease [10].

Conversely, extranodal marginal zone B-cell lymphoma of the mucosa-associated lymphoid tissue (MALT lymphoma) is the most common lymphoma subtype associated with SS. This variant of lymphoma commonly affects the salivary glands and is associated with a good prognosis. This striking association of MALT lymphomas, particularly involving the parotid gland, has been further elaborated in numerous clinical studies [54, 55]. A 28-fold increase in risk of MALT lymphoma was described in a population based case control study conducted by Smedby and colleagues [7]. On the other hand, when clinical features at presentation were evaluated in 33 patients with parotid MALT lymphoma, 46% of the patients had underlying SS [56]. Although an indisputable association already exists between SS and MALT lymphoma, a large number of DLBCL subtypes have also been reported in these patients [54, 55]. DLBCL in patients with pSS may be de novo or a result of transformation of previously unrecognized low grade MALT lymphoma [57]. Development of overt MALT lymphoma starts with early unremitting antigenic autoimmune stimulation, clonal expansion of B cells, and acquisition of genetic aberrations [58, 59]. Probable mechanism entails sequential progression from initial lymphoepithelial sialadenitis to acquirement of ectopic MALT tissue and further progressing to overt lymphoma.

Both cases of HL and NHL have been reported among SLE patients. However, the most common NHL subtype reported in these patients is DLBCL [60]. Bernatsky et al. reported a similar finding in a multicenter international cohort study [61]. Stimulated lymphocytes play a role in the activated B-cell subtypes of DLBCL, further reiterating the notion that chronic inflammation might heighten lymphoma risk in diseases like SLE [60]. A potential explanation for increased risk of NHL in SLE is that discrete major histocompatibility complexes (MHC) haplotypes may predispose to both disorders [62]. Another plausible mechanism is a defective immune system resulting in abnormal B-cell activation due to chronic and persistent antigenic stimulation, cell-cycle deregulation, and impaired apoptosis, ultimately resulting in uninhibited cell proliferation, heightened humoral immune response, and increased risk of oncogene translocation [63]. The impaired immune response in SLE is characterized by accumulation of activated self-reactive B and T cells [64]. Studies evaluating the risk of NHL in psoriasis patients have reported an increased risk of T-cell lymphoma overall or mycosis fungoides [46, 47, 65].

## 5. Disease Specific Risk Factors of Lymphomas

### 5.1. Rheumatoid Arthritis (RA) Associated Lymphomas

Increased risk of both NHL and HL has been reported in association with RA. There are however studies that have demonstrated variation in risk among RA patients. The strongest evidence indicating the correlation between disease activity and occurrence of lymphoma in RA patients was demonstrated by Baecklund and associates in a Swedish case control study [4]. 378 RA cases with lymphoma were compared to 378 RA patients in a control group without lymphomas. Information on disease characteristics such as number of swollen and tender joints, ESR values, and physician's global assessment score was obtained from medical records along with the course of treatment from onset of RA till lymphoma development. 70-fold increased lymphoma risk was observed in those RA patients with the highest disease activity as determined by the clinical components mentioned above. This finding was further corroborated in a smaller cohort study evaluating 29 RA patients with lymphoma [15]. It was further concluded that it was high inflammatory activity among RA patients, rather than its treatment, that was the major determinant in the development of lymphoma [4]. Indicators of disease severity such as presence of Felty's syndrome, high erythrocyte sediment rate (ESR) values, and erosive joint disease have been coupled with lymphoma risk in RA patients [66, 67].

### 5.2. Sjögren's Syndrome (SS) Associated Lymphomas

Increased occurrence of NHL has been reported in patients with Sjögren's syndrome, particularly primary Sjögren's syndrome (pSS). Sjögren's syndrome is characterized by lymphocytic infiltration of exocrine glands. In a cohort study evaluating risk of NHL in SS, 676 patients with pSS and 709 patients with secondary Sjögren's syndrome were enrolled. A relative risk of 8.7 (95% CI: 1.5–11) and 4.5 (95% CI: 1.5–11) was found in patients with primary and secondary form, respectively [68]. Numerous cohort and population based studies have established risk factors for Sjögren's syndrome associated lymphoma. Theander and colleagues proposed an increased risk of lymphoma in patients with follow-up period longer than 10 years [69]. In about 20–40% of the patients, the disease extends beyond exocrine glands. Epithelial lymphocytic invasion of the lung, liver, and kidneys or even immune complex mediated phenomena are evident in these patients. This subset of patients presents with major salivary gland enlargement and other features suggestive of extraglandular involvement such as lymphadenopathy, splenomegaly, peripheral neuropathy, and cutaneous lesions secondary to vasculitis [55, 69–71], all of which have been implicated with a higher risk of non-Hodgkin's lymphoma.

Lab findings such as cryoglobulinemia, low complement levels along with lymphopenia, and M components in serum or urine have also been suggested to contribute to higher risk of lymphomas [72]. Within the past 5 years, findings of severe glandular dysfunction, as identified by parotid scintigraphy, and ectopic germinal center structures, identified in minor salivary gland biopsies, at time of pSS diagnosis have also been proposed to play a role in lymphomagenesis [73, 74]. Similarly, in an isolated study evaluating the role of CD4 cytopenia and low CD4+/CD8+ ratio, these factors were the dominant predictors of lymphoma risk [69]. A bulk of lymphomas occurring in pSS are extranodal marginal zone lymphomas of mucosa-associated tissue (MALT lymphomas), along with some cases of DLBCL. Immunosuppressive therapy associated lymphoma risk has been rarely reported in patients with SS [75].

### 5.3. Systemic Lupus Erythematosus (SLE) Associated Lymphomas

Numerous cohort and case control studies have reported variable risk of lymphoma among SLE patients. In a Swedish population based cohort study with 5715 hospitalized SLE patients, there was nearly a 3-fold increase (SIR = 2.86, CI 95%: 1.96–4.04) in the incidence of NHL during the observation period [38]. Similarly, in an international study, a cohort of SLE patients from 23 centers were followed up for an average of 8 years [76]. This study included 9547 patients with SLE. The data gathered revealed a 3- to 4-fold increase in risk of NHL (3.64, 95% CI: 2.63–4.93). Disease related risk factors evaluated thus far include mean disease duration at the time of NHL diagnosis and disease severity. A mean disease duration of 12.4 years at diagnosis of NHL was also reported [77]. In a similar study, a mean duration of 17.8 years was reported by King and Costenbader [78]. Bernatsky and colleagues also identified a higher risk of SLE related lymphoma in males compared to female patients and this risk increased with older age. However, this finding was of no particular significance as these parameters were not different from those of the high risk group in the general population without SLE. Indicators of disease severity such as hematologic complications, involvement of the lungs, and sicca syndromes along with high SLE damage score have been implicated as predictors of lymphomas among this population [78–80]. On the other hand, studies have also demonstrated a relationship between exposure to medication and lymphoma development in these patients. A higher lymphoma risk in patients with exposure to cyclophosphamide and high cumulative steroids was seen in multisite SLE cohort analyses [77]. Alternatively, other studies reported negligible risk of treatment associated lymphomas in SLE patients [37]. Hence, role of immunosuppressive drugs in SLE related lymphoma does not seem to be crucial.

### 5.4. Polymyositis and Dermatomyositis Associated Lymphomas

Inflammatory myositis, particularly dermatomyositis, has been associated with a significant overall risk of malignancies. Lymphomas are among malignancies reported in this population. In the past, certain studies have reported up to 2- to 4-fold elevated risk of NHL in dermatomyositis patients [44, 81], while others failed to reveal any such association [45]. It was also concluded that the highest risk of malignancy in both polymyositis and dermatomyositis was at the time of diagnosis [44]. In a study conducted by Hill et al., four out of five NHL cases were diagnosed within the first year of follow-up or immediately preceding the diagnosis of myositis [44]. This in turn raises the question of whether the incidence of malignant lymphomas in these patients is a result of underlying inflammatory myositis or whether inflammatory myositis is merely a paraneoplastic phenomenon of underlying malignancy. More epidemiologic studies are needed to further clarify this association, as well as evaluate treatment related risk of lymphomas among these patients.

### 5.5. Spondyloarthropathies Associated Lymphomas

There is scarcity of data linking overall increased risk of lymphoproliferative malignancies with spondyloarthropathies, unlike RA and Sjögren's syndrome. Conflicting views have been put forth about direct association between lymphomas and psoriasis. However, most studies evaluating the risk of NHL in psoriasis patients have reported an increased risk of T-cell lymphoma overall or mycosis fungoides [46, 47, 65]. Similarly, Gelfand et al. reported an increase in risk of both HL and NHLs in 150,000 patients with mild psoriasis and 4000 patients with severe psoriasis [82]. A higher risk was seen in patients with severe disease (above 10-fold) when compared to the patients with milder disease (4-fold). However, other studies have failed to show such an association. This discrepancy in the number of lymphoma cases reported is possibly due to potential inflation of risk in some studies as T-cell lymphomas may often mimic psoriasis and hence may be misclassified.

One other spondyloarthropathy linked to risk of lymphomagenesis is ankylosing spondylitis. Although most studies have not revealed a significant link between AS and lymphomas, Mullighan et al. reported almost a 3-fold higher risk of lymphoma (SIR 2.8; 95% CI: 1.4–5.6) [83]. Despite few cases of positive association between AS and lymphomas, this connection can be refuted due to scarcity of data and lower magnitude of such relationship when compared to lymphoma risk in RA or SS.

## 6. Therapy Related Risk of Lymphomas 

It has been debated for years whether immunosuppressive medication used to treat inflammatory diseases could carry an innate and subsequently amplified risk of lymphoma. These drugs include widely used disease modifying antirheumatic drugs (DMARDs) such as methotrexate (MTX) and azathioprine and biologic agents such as infliximab and etanercept. Several studies have explored the association between lymphoma and RA therapy (Table 3). However, inconsistent results have been reported in these studies. Incidence of MTX-LPD was assessed in 403 RA patients in a study conducted by Yoshida et al. [84]. Four out of the 403 RA patients being treated with MTX developed lymphomas. The patients who developed MTX-LPDs had significantly shorter disease duration and over half of them showed tumor regression following cessation of MTX [84], therefore highlighting the possibility of MTX related lymphomagenesis. Similar finding of MTX-LPD regression following treatment cessation was reported by Ikeda and associates in a rare case of gastric lymphoma in a RA patient [85]. Another noteworthy finding is the involvement of extranodal sites in 40–50% of MTX related LPD cases. Extranodal sites include gastrointestinal tract, skin, liver, lung, and kidney. In addition, several cases of MTX related EBV associated LPD have also been documented. It is a well recognized fact that patients with immunodeficiency, such as those on MTX therapy, have a high risk of developing lymphoma, and EBV is associated with pathogenesis of lymphomas. Approximately 30–50% of lymphomas in RA patients on MTX therapy are EBV positive [86, 87]. However, the precise role of methotrexate in lymphomagenesis is poorly understood and is yet to be validated. For example, several of these studies have not taken into account the effect of underlying disease, disease severity, and reasons for treatment. Most larger studies have reported negligible risk of lymphoma in RA patients on chronic MTX therapy [88]. The strong association between disease severity and propensity for antirheumatic treatment makes it hard to understand whether the disease severity and underlying pathology themselves contribute to lymphomagenesis or whether they are actually treatment related. Similarly, an association between azathioprine and lymphoma has been reported in patients with autoimmune diseases. However, a meta-analysis conducted by Kandiel et al. suggested a stronger role of underlying disease and a combination of factors with lymphomagenesis when compared to role of azathioprine [89].

Other non-DMARD therapies such as use of NSAIDs and systemic corticosteroids have also been suspected to play a role in lymphomagenesis. However, several cohort and case control studies revealed no significant association between use of NSAIDs and steroids and lymphoma risk. TNF antagonists are a commonly prescribed group of medications in RA patients. A number of cases of lymphomas were documented in several patients in the earlier trials of this class of medication [90, 91]. However, meta-analysis of observational data from registries as well as randomized controlled trials has failed to show a significant increase in the risk of lymphoma especially if a sufficiently large sample size was enrolled in the study with adequate follow-up period [92]. The current literature evaluating the role of medication related lymphomas in RA offers minimal insight secondary to limitations by potential confounding.

## 7. Conclusion

Link between autoimmune diseases and lymphomas has been validated by several population based studies in the past. In particular, studies exploring RA and Sjögren's syndrome associated lymphomas have brought forth a great deal of evidence to light. On the other hand, it still remains ambiguous why other autoimmune conditions, such as type 1 diabetes mellitus, multiple sclerosis, and sarcoidosis, do not present an amplified risk of lymphoma. Studies exploring risk of lymphoma in multiple sclerosis and diabetes mellitus have reliably shown no excess risk of lymphoma or hematologic malignancy [93–96]. A few cases of Hodgkin's lymphoma have been reported in patients with sarcoidosis. A large cohort study of 9000 sarcoidosis patients reported a twofold increased risk of lymphoma overall (SIR 1.9; 95% CI: 1.3–2.7), with significantly increased risk of lymphoma confined to the initial four years of follow-up [97]. However, two other smaller cohort studies did not report such an association [98, 99]. Therefore, although the strength of association between increased risk of lymphoma and sarcoidosis remains weak, it cannot be completely excluded due to inadequate evidence.

Among patients with autoimmune arthritis, severity of disease and higher degree of inflammation have consistently been associated with increased risk of lymphoma. Another noteworthy fact is the display of different spectra of lymphomas in each variant of autoimmune arthritis, further emphasizing the notion that lymphomagenesis is probably disease specific. As mentioned earlier, DLBCL has been linked to both SLE and RA. This could potentially be a result of chronic activation of peripheral B cells leading to uncontrolled proliferation of clonal B-cell populations, ultimately resulting in a lymphoma. Also worthy of note, autoimmune disease associated lymphomas are overwhelmingly of B-cell type, the major exceptions being the intestinal T-cell lymphomas that arise in long-standing celiac disease and in association with infection with the retrovirus HTLV-1. The higher occurrence of B-cell type lymphomas in autoimmune diseases can perhaps be explained by the role of B cells at a cellular level. Functions of B cells include secretion of autoantibodies, autoantigen presentation, reciprocal interactions with T cells, secretion of inflammatory cytokines, and generation of ectopic germinal centers [100]. Through these mechanisms, B cells are involved both in pathogenesis of autoimmune diseases and in lymphomagenesis.

Alternate risk factors for lymphomagenesis include exposure to infectious agents and environmental factors such as smoking in addition to genetic susceptibility. Infectious agents linked to lymphomagenesis include Epstein-Barr virus (EBV), human immunodeficiency virus (HIV), hepatitis C virus (HCV), and human T-cell lymphotropic virus-1 (HTLV-1). Among these infectious agents, EBV and HCV are of particular interest. EBV demonstrates oncogenic potential by persisting in B cells and potentially transforming these cells. Several case reports have proposed a potential role of EBV in lymphomagenesis in patients with MTX-LPD among RA patients. Spontaneous regression of EBV positive lymphomas was witnessed in these cases following cessation of MTX therapy for RA. However, no increase in distribution of EBV positive lymphomas was seen in RA patients when compared to the lymphomas arising in the general population. HCV infection has also been implicated in the development of B-cell NHL. Pathogenic processes accountable for HCV related lymphoproliferative disorders remain ambiguous. Existing evidence supports a model in which chronic stimulation of B cells by antigens associated with HCV infection causes nonmalignant cell expansion that may evolve into B-cell NHL. Probable interactions between HCV and other viral agents such as HIV and EBV have also been investigated in development of lymphoproliferative disorders [101]. HCV has been found in a large proportion of salivary gland MALT lymphomas, particularly in areas endemic to this virus [56, 102].

Smoking is an independent risk factor in both lymphomas and autoimmune/inflammatory arthritis. Past studies have also linked smoking to specific variants of lymphomas. These variants include follicular lymphoma, T-cell lymphoma, and Hodgkin's lymphoma. Gibson et al. reported a 30% increase in risk of follicular lymphoma but not non-Hodgkin's lymphoma (NHL) overall or other NHL subtypes [103, 104]. Similarly, a relationship between smoking and amplified risk of T-cell lymphoma and Hodgkin's lymphoma has been suggested by Sergentanis and colleagues [105]. Previous literature has also shown an association between smoking and anti-citrullinated protein antibody-positive RA and SLE [106]. However, there is shortage of data linking smoking to RA associated lymphomas. This remains an area for further exploration in future studies. An alternative hypothesis linking RA and lymphoma risk is genetic susceptibility, along with environmental risk factors. In a registry based cohort study, evaluating the risk of lymphoma in family members of RA patients, there was marginal or no significant increase in risk of lymphoma among parents and siblings of the sample population [13]. These findings further corroborate the fact that increased lymphoma risk in RA patients is probably secondary to factors directly associated with disease or its treatment [13].

It remains unclear whether the clinical course of lymphomas complicating autoimmune arthritis is different from that of lymphomas occurring in the general population. In 2003, a Swedish study reported a median survival rate of 6 months in patients with RA/NHL [6]. On the other hand, a larger, more recent study of 65 RA lymphoma cases and 1500 non-RA lymphoma controls reported a hazard ratio of 0.60 (95% CI: 0.37–0.98) [107]. This disparity in results makes it difficult to truly understand the clinical outcome of autoimmune disease associated lymphomas. However, the disparity in outcomes of the two abovementioned studies can be attributed to the variation in sample size as well as to the advances in lymphoma related diagnostic and treatment modalities. One such example is the emerging role of rituximab, a monoclonal anti-CD20 antibody that can be used in the treatment of lymphomas and autoimmune arthritis. A true grasp on this matter can only be obtained in the future following a thorough investigation using large population based studies.

Autoimmune rheumatic diseases and lymphocytic malignancies exhibit a bidirectional relationship. It is important to differentiate lymphomas that occur in the course of an autoimmune disease from a lymphoma presenting with paraneoplastic multisystemic involvement or autoimmune rheumatic manifestations. Several patients present with rheumatic manifestations month to years after the onset of lymphocytic malignancy [108]. Currently, it is also pertinent to conduct detailed molecular studies to determine the key pathogenic events in lymphomagenesis. It is also imperative to identify “triggers” or risks for malignant lymphomas in this population. This would help define algorithms to identify autoimmune arthritis patients with higher likelihood of lymphomagenesis. Quantifying level of risk in the target population prior to initiation of treatment will help us better understand the role of immunosuppressive therapy in lymphomagenesis. Additional research is needed to definitively state whether therapy in arthritis contributes to risk of lymphomas or alters the clinical course of malignant lymphomas in these patients.

## Figures and Tables

**Table 1:** Studies exploring risk of lymphomas in patients with RA, SLE, Sjögren's syndrome, inflammatory arthritis, and spondyloarthropathies.

Author	Year	Country	Disease	Population (*n*/*N*)	Variant	RR/SIR/OR (CI)
Thomas et al. [11]	2000	Scotland	RA	17/26,623 10/26,623	HD (SIR) NHL	3.85 (2.2, 6.2) 2.13 (1.7, 2.6)
Mariette et al. [12] (TNFI)	2011	UK	RA	18/30,000	NHL	1.07 (0.6, 1.7)
Ekström et al. [13]	2003	Sweden	RA	77/76,527 18/30,000	HD NHL	3.06 (2.4, 3.8) 1.89 (1.7, 2.1)
Abásolo et al. [14]	2008	Southern Europe	RA	3/789	NHL	5.4 (1.1, 15.7)
Wolfe and Michaud [15]	2004	USA	RA	4/13,869 42/13,869	HD NHL	3.0 (1.3, 6.8) 1.7 (1.3, 2.2)
Hemminki et al. [16]	2008	Sweden	RA	35/42,262 280/42,262	HD NHL	4.05 (2.8, 5.6) 2.34 (2.1, 2.6)
Anderson et al. [17]	2009	USA	RA	1157 cases/3289 controls	NHL	OR 1.2 (1.1–1.3)
Askling et al. [18]	2009	Sweden	RA	26/26,981	HD	2.7 (1.8–4.1)
Parikh-Patel et al. [19]	2009	USA	RA	325/84,475	NHL - Men, women	2.1 (1.7, 2.5) 1.4 (1.2, 1.6)
Hellgren et al. [20]	2010	Sweden	RA	19 cases/53 controls	HD	OR 1.8 (1.0–3.0)
Chen et al. [21]	2010	Taiwan	RA	1/23,644 59/23,644	HD NHL	1.76 (1.5, 2.2) 3.54 (3.45, 3.63)
Mercer et al. [22]	2013	UK	RA	16/3771	NHL	3.12 (1.8, 5.1)
Dreyer et al. [23]	2013	Denmark	RA	5/3812	NHL	2.27 (0.9, 5.5)
Anderson et al. [17]	2009	USA	SS	142 cases/255 controls	NHL	1.9 (1.5–2.3)
Zhang et al. [24]	2010	China	SS	8/1320	NHL	48.1 (20.7–94.8)
Solans-Laqué et al. [25]	2011	Spain	SS	11/244	NHL	15.6 (8.7–28.2)
Weng et al. [26]	2012	Taiwan	SS	277/7852	NHL - Men NHL- Women	3.1 (0.6–9.0) 7.1 (4.2–10.3)
Johnsen et al. [27]	2013	Norway	SS	7/443	NHL	9 (7.1–26.3)
Liang et al. [28]	2014	Meta-analysis	SS	14 studies	Lymphomas	13.8 (8.5–19.0)
Feltelius et al. [29]	2003	Sweden	AS	12/6621	Lymphomas	1.3 (0.9–1.9)
Shibata et al. [30]	2004	Japan	AS	NA/3262	Lymphomas	2.8 (1.4–5.6)
Becker et al. [31]	2005	Germany	AS	710 cases/controls	Lymphomas	3.0 (0.1–29)
Askling et al. [32]	2006	Sweden	AS	50,615 cases/92,928 controls	Lymphomas	1.0 (0.6–1.7)
Mellemkjaer et al. [33]	2008	Denmark	AS	25,941 cases/58,551 controls	Lymphomas	1.1 (0.6–1.8)
Anderson et al. [17]	2009	USA	AS	33,721 cases/122,531 controls	Lymphomas	1.1 (0.7–1.5)
Rohekar et al. [34]	2008	Canada	PsA	NA/665	Lymphomas	0.7 (0.3–1.8)
Gross et al. [35]	2014	USA	PsA	3/2977	Lymphomas	0.4 (0.1–1.2)
Nived et al. [36]	2001	Sweden	SLE	2/116	NHL	11.63 (1.40, 42.0)
Cibere et al. [37]	2001	Canada	SLE	4/297	NHL	7 (1.9, 8)
Björnådal et al. [38]	2002	Sweden	SLE	32/5715	NHL	2.86 (1.96, 4.04)
Tarr et al. [39]	2007	Hungary	SLE	2/860	NHL	3.50 (0.4, 12.5)
Parikh-Patel et al. [40]	2008	USA	SLE	96/30478	NHL	2.74 (2.22, 3.34)
Anderson et al. [17]	2009	USA	SLE	129 cases/285 controls	NHL (OR)	1.5 (1.2–1.9)
Kang et al. [41]	2010	Korea	SLE	3/914	NHL	15.4 (2.9–37.7)
Chen et al. [21]	2010	Taiwan	SLE	NA/11763	Lymphomas	7.3 (7.0–7.6)
Dreyer et al. [42]	2011	Denmark	SLE	4/576	NHL	5.0 (1.9–13.3)
Bernatsky et al. [43]	2013	Scotland	SLE	76/16409	NHL HL	4.4 (3.5–5.5) 2.3 (0.9–4.7)
Hill et al. [44]	2001	Sweden, Denmark, and Finland	Dermatomyositis	NA/618	NHL	3.6 (1.2, 11.1)
Stockton et al. [45]	2001	Scotland	Dermatomyositis	2/50 1/50	NHL HL	13.3 (1.6, 48) 28.4 (0.7, 157.9)
Hill et al. [44]	2001	Sweden, Denmark, and Finland	Polymyositis	NA/914	NHL	3.7 (1.7–8.2)
Stockton et al. [45]	2001	Scotland	Polymyositis	2/40 2/40	NHL HL	5 (0.6, 18.1) 31 (3.8, 112)

RA: rheumatoid arthritis; SLE: systemic lupus erythematosus; AS: ankylosing spondylitis; SS: Sjögren's syndrome; PsA: psoriatic arthritis; RR: relative risk; SIR: standardized incidence ratio; OR: odds ratio; CI: confidence interval; *n*: number of cases of lymphoma; *N*: study population; NA: not available.

**Table 2:** Autoimmune arthritis specific lymphoma variants.

Disease	Associated lymphoma subtype	Reference
Rheumatoid arthritis (RA)	Diffuse large B-cell lymphoma (DLBCL)	[4, 6, 7]
Primary Sjögren's syndrome (pSS)	Mucosa-associated lymphoid tissue (MALT) Diffuse large B-cell lymphoma (DLBCLs)	[35–37]
Systemic lupus erythematosus (SLE)	Diffuse large B-cell lymphoma (DLBCL)	[38, 39]
Inflammatory myositis	No specific association	
Psoriasis	T-cell lymphoma Mycosis fungoides	[46, 47]

**Table 3:** Selective studies evaluating therapy related risk of lymphoma in RA patients.

Author	Year	Therapy	Disease	Sample population	Measure of risk	Comparison group	Risk
Wolfe and Michaud [48]	2007	Inf., Etan., and Ana.	RA	19,591	OR	All RA patients not on anti-TNF therapy	1.0 (0.6, 1.8) *P* = 0.875
		Anti-TNF therapy plus MTX	RA		OR	MTX therapy alone	1.1 (0.6, 2.0) *P* = 0.710

Geborek et al. [49]	2005	Inf. and Etan.	RA	757 (anti-TNF group) versus 800 (conventional treatment)	AHR	Anti-TNF alpha naive RA patients	5.0 (0.9, 27.9) *P* = 0.06

Askling et al. [50]	2005	Inf., Etan., and Ana.	RA	4160	ARR	All RA patients/all treatments	1.1 (0.6, 2.1)

Setoguchi et al. [51]	2006	Inf., Etan., and Ana.	RA	1152 biologic users versus 7306 MTX users	AHR	RA patients with MTX use only	1.11 (0.51, 2.37)

Askling et al. [18]	2009	Inf., Etan., and Ana.	RA	6604	ARR	Anti-TNF alpha naive RA patients	1.35 (0.82, 2.11)

Wolfe and Michaud [52]	2004	MTX	RA	18,572	SIR	General population	1.7 (95% CI 0.9–3.2)
		Infliximab and etanercept			SIR		2.9 (95% CI 1.7–4.9)

Buchbinder et al. [53]	2008	MTX	RA	459	SIR	General population	5.1 (2.2–10.0)

Inf.: infliximab; Etan.: etanercept; Ana.: anakinra; RA: rheumatoid arthritis; MTX: methotrexate; ARR: adjusted relative risk; AOR: adjusted odds ratio; AHR: adjusted hazard ratio; SIR: standardized incidence ratio.
